# Epidemiology of acute kidney injury in the clinical emergency: A prospective cohort study at a high-complexity public university hospital in São Paulo, Brazil

**DOI:** 10.1371/journal.pone.0309949

**Published:** 2024-09-05

**Authors:** Flávia Barros de Azevedo, Farid Samaan, Dirce Maria Trevisan Zanetta, Luis Yu, Irineu Tadeu Velasco, Emmanuel de Almeida Burdmann

**Affiliations:** 1 Division of Clinical Emergencies, Hospital University of São Paulo, São Paulo, São Paulo, Brazil; 2 Mobile Emergency Care Service, Porangatu, Goias, Brazil; 3 Planning and Evaluation Group, São Paulo State Department of Health, São Paulo, São Paulo, Brazil; 4 Research Division, Dante Pazzanese Institute of Cardiology, São Paulo, São Paulo, Brazil; 5 School of Public Health, University of São Paulo, São Paulo, São Paulo, Brazil; 6 Laboratório de Investigação Médica (LIM) 12, Serviço de Nefrologia, Faculdade de Medicina, Universidade de São Paulo, São Paulo, Brazil; Azienda Ospedaliero Universitaria Careggi, ITALY

## Abstract

**Introduction:**

Southern Hemisphere countries have been underrepresented in epidemiological studies on acute kidney injury (AKI). The objectives of this study were to determine the frequency, risk factors, and outcomes of AKI in adult hospitalized patients from the emergency department of a public high-complexity teaching hospital in the city of São Paulo, Brazil.

**Methods:**

Observational and prospective study. AKI was defined by the KDIGO guidelines (Kidney Disease: Improving Global Outcomes) using only serum creatinine.

**Results:**

Among the 731 patients studied (age: median 61 years, IQR 47–72 years; 55% male), 48% had hypertension and 28% had diabetes as comorbidities. The frequency of AKI was 52.1% (25.9% community-based AKI [C-AKI] and 26.3% hospital-acquired AKI [H-AKI]). Dehydration, hypotension, and edema were found in 29%, 15%, and 15% of participants, respectively, at hospital admission. The in-hospital and 12-month mortality rates of patients with vs. without AKI were 25.2% vs. 11.1% (p<0.001) and 36.7% vs. 12.9% (p<0.001), respectively. The independent risk factors for C-AKI were chronic kidney disease (CKD), chronic liver disease, age, and hospitalization for cardiovascular disease. Those for H-AKI were CKD, heart failure as comorbidities, hypotension, and edema at hospital admission. H-AKI was an independent risk factor for death in the hospital, but not at 12 months. C-AKI was not a risk factor for death.

**Conclusions:**

AKI occurred in more than half of the admissions to the clinical emergency department of the hospital and was equally distributed between C-AKI and H-AKI. Many patients had correctable risk factors for AKI, such as dehydration and arterial hypotension (44%) at admission. The only independent risk factor for both C-AKI and H-AKI was CKD as comorbidity.

## Introduction

Acute kidney injury (AKI) is a global public health problem due to its high incidence, its short- and long-term morbidity and mortality, the damage it does to the quality of life of survivors, and the great impact on the consumption of human and economic resources for its management [1–5]. The increased observed incidence of AKI is likely due to the increase in the number of patients diagnosed by the new definitions [6], the greater longevity of people (with a consequent increase in chronic multimorbidity) and greater access to complex treatments [1, 5]. The new international consensus for the definition, classification, and management of AKI [7, 8], and the global initiatives to alert health providers and managers about the relevance of this syndrome, have been essential to improve the identification and treatment of AKI [9–11].

Demographic and epidemiological transitions face a special challenge for preventing, diagnosing, and treating AKI [12]. Brazil stands out as an upper-middle-income Latin American nation in which the public, universal Unified Health System is primarily responsible for providing health care to more than 150 million people [13]. In in the last two decades, the percentage of Brazilians who are over 60 years of age has increased from 9% to 15% [14]. In the same period, the prevalence of arterial hypertension and diabetes mellitus in the Brazilian population increased from 21% to 25% and from 5% to 9%, respectively [15].

Brazilian studies on AKI conducted during the COVID-19 pandemic were valuable for showing the severity and care gaps related to AKI treated with kidney replacement therapy (KRT) [16, 17]. On the other hand, detailed epidemiological information on AKI, derived from prospective studies in patients admitted to the emergency department is scarce in Latin America. The objectives of this study were to determine the frequency, risk factors, and outcomes of AKI in adult hospitalized patients from the emergency department of a public high-complexity teaching hospital in the city of São Paulo, Brazil.

## Methods

### Design, study site, and inclusion and exclusion criteria

This study was based on an observational, prospective database of patients who required admission from the clinical emergency department of a public, high-complexity teaching hospital. The recruitment period for this study was between July 1, 2015, and September 30, 2017. The inclusion criteria were age ≥18 years and hospitalization starting at the time of clinical emergency. Patients who underwent chronic outpatient KRT before admission, those who were hospitalized due to emergency KRT or who received exclusive palliative care were excluded from the study. The participants provided written informed consent, and the study was approved by the Research Ethics Committee of the University of São Paulo School of Medicine Hospital das Clínicas under number CAAE 38527814.0.3001.5421.

### Variables and definitions

Demographic data (age, sex) and information on the presence or absence of the following comorbidities were obtained: arterial hypertension (self-reported or use of antihypertensive drugs), diabetes mellitus (self-reported or use of oral antidiabetic agents or insulin), heart failure (reported from medical records or echocardiograms showing left ventricular ejection fraction <40%), coronary artery disease (history of angina, acute myocardial infarction, coronary stent placement, or coronary artery bypass graft surgery), cerebrovascular disease (history of transient ischemic attack or stroke), chronic kidney disease (self-reported), chronic obstructive pulmonary disease (history of asthma, chronic bronchitis, or pulmonary emphysema), chronic liver disease (history of liver cirrhosis or chronic, autoimmune, or alcoholic viral hepatitis), and neoplasia (history of cancer). The household use of the following medications was assessed: angiotensin-converting enzyme inhibitors (ACEIs), angiotensin receptor blockers (ARBs), and nonsteroidal anti-inflammatory drugs (NSAIDs).

The presence or absence of dehydration, edema, and arterial hypotension (systolic arterial pressure <90 mmHg) were evaluated at hospital admission. The main cause of hospitalization was recorded according to the chapters of the International Classification of Diseases, 10th revision (ICD-10) [18]. Serum creatinine was collected at admission and daily up to the first seven days of hospitalization. The glomerular filtration rate (GFR) at admission was estimated using the CKD-EPI equation (Chronic Kidney Disease Epidemiology Collaboration) 2009 [19]. Hospital-acquired AKI (H-AKI) was defined by an increase in serum creatinine by ≥0.3 mg/dl within 48 hours or increase in serum creatinine to ≥1.5 times within seven days [7]. Community-acquired AKI (C-AKI) was defined by decrease in serum creatinine by ≥0.3 mg/dl within 48 hours or a decrease in serum creatinine to ≥1.5 times within r seven days [9, 20]. H-AKI was classified as stage 1 (increase in serum creatinine up to 1.9 times the baseline value), stage 2 (increase to 2.0–2.9 times the baseline value), or stage 3 (increase to more than 2.9 times the baseline value) [7]. The following additional data were obtained: use or absence of antimicrobials, nephrotoxic drugs (aminoglycosides, nonsteroidal anti-inflammatory drugs, antiretroviral drugs, iodinated contrast agents, polymyxin, chemotherapeutic agents, vancomycin), need for vasopressors, and need for mechanical ventilation during hospitalization. The outcomes of interest were AKI, need for KRT, length of hospital stay, need for intensive care, and in-hospital and 12-month mortality.

### Statistical analysis

Categorical variables are described as frequencies. The normality of the distribution of quantitative variables was assessed using the Kolmogorov–Smirnov test. Normally distributed quantitative variables are expressed as mean ± standard deviation, and nonnormally distributed variables are expressed as median (interquartile range (IQR)). Frequencies were compared by the χ^2^ test or Fisher’s exact test, as appropriate. Intergroup comparisons of quantitative variables were done with Student’s t test and the Mann–Whitney U test for normally distributed and nonnormally distributed data, respectively.

Multiple logistic regression analysis was performed to analyze the risk factors for C-AKI, H-AKI, and mortality. The models were constructed using backward stepwise selection. When there was a change in the estimate of a parameter greater than 10% with the exclusion of a variable, that variable was kept in the model for adjustment. The goodness of fit was assessed with the Hosmer–Lemeshow test, and the significance of the variables was assessed using the Wald test.

To analyze factors associated with C-AKI, the initial model included the following variables: age (continuous variable), diabetes mellitus (DM), chronic kidney disease (CKD), chronic liver disease, use of ACEIs or ARBs, use of NSAIDs and hospitalizations for diseases of the cardiovascular and genitourinary systems. The statistical model for the analysis of factors associated with H-AKI included the variables age (continuous variable), sex, arterial hypertension (AH), DM, heart failure (CHF), chronic liver disease, CKD, mean arterial pressure (MAP) <65 mmHg, dehydration, and edema. For the analysis of factors associated with in-hospital death, the model included the following variables: age (continuous variable); sex; hospitalization for respiratory, hepatobiliary, and genitourinary diseases; MAP <65 mmHg; dehydration; estimated glomerular filtration rate (eGFR) at admission <60 ml/min/1.73 m^2^; community-acquired AKI and AKI (C-AKI); use of vasopressors; need for mechanical ventilation; and ICU admission. The following variables were used to estimate the 12-month risk of death among survivors of hospitalization: age (continuous variable), sex, CHF status, chronic liver disease status, hospitalization for respiratory and genitourinary diseases, dehydration status, eGFR at admission. <60 ml/min/1.73 m^2^, use of vasopressors, need for mechanical ventilation, ICU admission, H-AKI, and C-AKI. Statistical analysis was performed with SPSS software, version 19.0 (SPSS Inc., Chicago, IL, USA). The threshold for significance was <0.05, and 95% confidence intervals were calculated.

## Results

**Fig 1:**
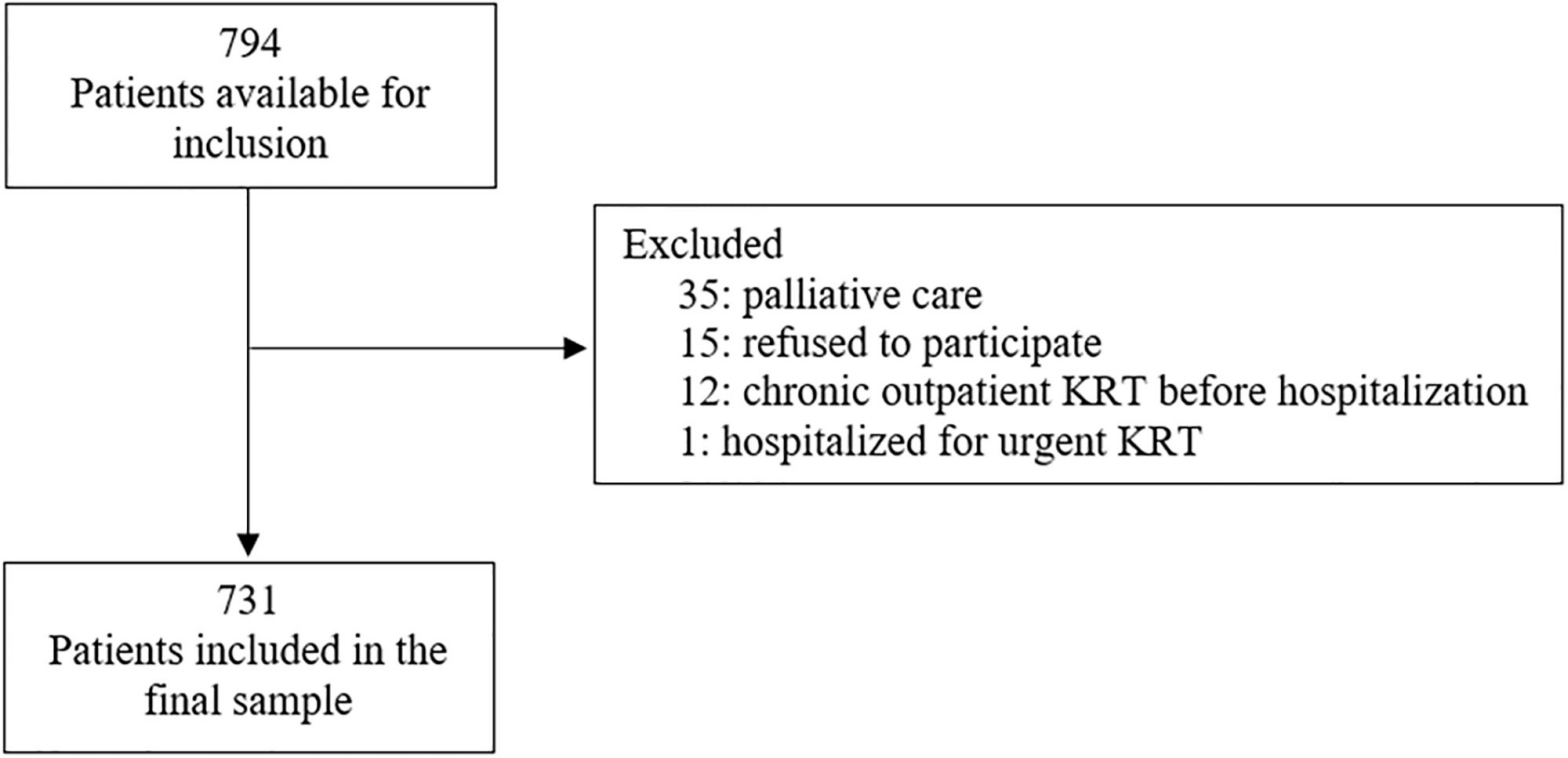
Flowchart of patient inclusion.

During the study period, 794 patients admitted to the emergency department were evaluated. After excluding individuals in palliative care (35), patients who refused to participate (15), patients who were on chronic outpatient KRT before admission (12), and patients who were hospitalized due to emergency KRT (1), 731 patients were included in the final sample (Fig 1).

### Overall characteristics of the studied population

The median age was 60.6 (46.7–71.9) years, and there was a predominance of males (54.9%). The main comorbidities were hypertension (47.7%), DM (27.5%), CHF (23.3%), chronic liver disease (17.0%), COPD (16.8%), cerebrovascular disease (10.7%), and CKD (10.4%). The most frequent causes of hospitalization, according to the recorded ICD-10 codes, were respiratory diseases (31.7%), genitourinary diseases (14.8%), cardiovascular diseases (10.7%), infections/septicemia (10.1%), and diseases of the liver and bile duct (8.5%). At hospital admission, dehydration, hypotension, and edema were present in 29.4%, 15.0%, and 14.5% of the patients, respectively. During hospitalization, 16.0% of patients used nephrotoxic drugs, and 71.4% used antimicrobial agents. C-AKI, H-AKI, and AKI requiring KRT occurred in 25.9%, 26.3%, and 3.3% of the patients, respectively. The total frequency of AKI was 52.1%. The need for intensive care was 24.9%, the need for mechanical ventilation was 18.1%, and the need for vasopressors was 14.9%. The in-hospital and 12-month mortality rates were 18.5% and 26.0%, respectively (Table 1).

**Table 1:** Demographic profile, comorbidities, and outcomes of the participants.

Variable	All (n = 731)
Demographic data	
Age, years	60.6 (46.7–71.9)
Male sex, % (n)	54.9 (401)
Comorbidities	
Arterial hypertension, % (n)	47.7 (349)
Diabetes mellitus, % (n)	27.5 (201)
Heart failure, % (n)	23.3 (170)
Chronic liver disease, % (n)	17.0 (124)
Chronic lung disease, % (n)	16.8 (123)
Neoplasm, % (n)	13.8 (101)
Cerebrovascular disease, % (n)	10.7 (78)
Known chronic kidney disease, % (n)	10.4 (76)
Coronary artery disease, % (n)	9.6 (70)
Number of comorbidities	2 (1–3)
Causes of hospitalization according to ICD chapter	
Respiratory system, % (n)	31.7 (232)
Genitourinary system, % (n)	14.8 (108)
Cardiovascular system, % (n)	10.7 (78)
Infection/sepsis, % (n)	10.1 (74)
Liver/bile duct diseases, % (n)	8.5 (62)
Skin and subcutaneous tissue, % (n)	5.6 (41)
Endocrine/metabolic, % (n)	3.6 (26)
Hematological, % (n)	2.6 (19)
Neoplasm, % (n)	2.5 (18)
Rheumatological, % (n)	2.5 (18)
Others, % (n)	7.5 (55)
Parameters at hospital admission	
Systolic blood pressure (mmHg)	120 (100–130)
Diastolic blood pressure (mmHg)	70 (60–80)
Arterial hypotension, % (n)	15.0 (110)
Dehydration, % (n)	29.4 (215)
Edema, % (n)	14.5 (106)
Serum creatinine (mg/dl)	0.75 (0.98–1.44)
eGFR (ml/min/m^2^)	72.4 (44.5–99.2)
eGFR < 60 ml/min/m^2^, % (n)	38.6 (282)
Previously used medications	
ACEI or ARB, % (n)	27.0 (193)
Nonsteroidal anti-inflammatory drugs, % (n)	1.5 (9)
Medications used during hospitalization	
Antimicrobials, % (n)	71.4 (522)
Nephrotoxic, % (n)	16.0 (117)
Vasopressors, % (n)	14.9 (109)
Need for mechanical ventilation, % (n)	18.1 (132)
Renal outcomes	
AKI, % (n)	52.1 (381)
C-AKI, % (n)	25.9 (189)
H-AKI, % (n)	26.3 (192)
Need for dialysis, % (n)	3.3 (24)
Nonrenal outcomes	
Need for ICU, % (n)	24.5 (179)
Length of hospital stay (days)	9 (4–15)
In-hospital death, % (n)	18.5 (135)
Death at 12 months, % (n)	26.0 (180)

Data are presented as % (n) or median (IQR). eGFR, estimated glomerular filtration rate. ACEI, angiotensin-converting enzyme inhibitor. ARB, angiotensin receptor blocker. Arterial hypotension, blood pressure <90 mmHg. AKI, acute kidney injury. C-AKI, community-acquired AKI. H-AKI, hospital-acquired AKI. ICU, intensive care unit.

### Comparing AKI and non-AKI groups

The AKI patients were older [62.0 (50.0–73.1) vs. 58.0 (41.3–70.8) years, p = 0.02], had more comorbidities [2 (IQR 1–3) vs. 1 (IQR 0–2), p<0.001], and had a higher proportion of patients hospitalized for diseases of the urinary tract than the non-AKI group [18.4% vs. 10.9%, p = 0.019]. Patients with AKI were more likely to have hypotension [18.4% vs. 11.4%, p = 0.009], dehydration [35.2% vs. 23.1%, p<0.001], or edema [17.8% vs. 10.9%, p = 0.007] at admission than no-AKI patients. During hospitalization, patients with AKI used more frequently vasopressors [21.3% vs. 8.0%, p<0.001], mechanical ventilation [25.2% vs. 10.3%, p<0.001], and were admitted to the ICU more frequently [32.8% vs. 15.4%, p<0.01]. In-hospital death was higher in patients with AKI [25.2% vs, 11.1%, p<0.001]. There was no difference in 12-month mortality between patients with and without AKI [15.4% versus 12.9%, respectively, p = 0.366] (Table 2).

**Table 2:** Comparison between patients without and with acute kidney injury.

Variable	No AKI (n = 350)	AKI (n = 381)	p value
Age, years	**58.0 (41.3–70.8)**	**62.0 (50.0–73.1)**	**0.002**
Male sex, % (n)	54.9 (192)	54.9 (209)	1.000
Comorbidities			
Arterial hypertension, % (n)	**43.1 (151)**	**52.0 (198)**	**0.017**
Diabetes mellitus, % (n)	24.3 (85)	30.4 (116)	0.062
Heart failure, % (n)	**18.0 (63)**	**28.1 (107)**	**0.001**
Chronic liver disease, % (n)	**13.4 (47)**	**20.2 (77)**	**0.015**
CKD, % (n)	**5.4 (19)**	**15.0 (57)**	**<0.001**
Number of comorbidities	**1 (0–2)**	**2 (1–3)**	**<0.001**
Causes of hospitalization			
Respiratory system diseases, % (n)	32.6 (114)	31.0 (118)	**0.019**
Diseases of the genitourinary tract, % (n)	**10.9 (38)**	**18.4 (70)**	
Diseases of the cardiovascular system, % (n)	12.3 (43)	9.2 (35)	
Infection/sepsis, % (n)	9.1 (32)	11.0 (42)	
Liver/bile duct diseases, % (n)	7.1 (25)	9.7 (37)	
Previously used medications			
ACEI or ARB, % (n)	23.8 (81)	29.9 (112)	0.062
Nonsteroidal anti-inflammatory drugs, % (n)	1.7 (5)	1.3 (4)	0.657
Physical examination on admission			
Arterial hypotension, % (n)	**11.4 (40)**	**18.4 (70)**	**0.009**
Dehydration, % (n)	**23.1 (81)**	**35.2 (134)**	**<0.001**
Edema, % (n)	**10.9 (38)**	**17.8 (68)**	**0.007**
Use of vasopressors, % (n)	**8.0 (28)**	**21.3 (81)**	**<0.001**
Need for mechanical ventilation, % (n)	**10.3 (36)**	**25.2 (96)**	**<0.001**
Nonrenal outcomes			
Need for ICU, % (n)	**15.4 (54)**	**32.8 (125)**	**<0.001**
Length of stay (days)	**6 (4–13)**	**10 (5–17)**	**<0.001**
In-hospital death, % (n)	**11.1 (39)**	**25.2 (96)**	**<0.001**
Death at 12 months, % (n)	**12.9 (40)**	**36.7 (140)**	**<0.001**

Data are presented as % (n) or median (IQR). AKI, acute kidney injury. C-AKI, community-acquired AKI. H-AKI, hospital-acquired AKI. CKD, chronic kidney disease. ICU, intensive care unit. The numbers in bold indicate column values that differ from each other within the same row at the 0.05 significance level.

### Comparing H-AKI and C-AKI groups

Compared with patients with H-AKI, those with C-AKI had a higher frequency of hypotension [24.3% vs. 12.5%, p = 0.003] and dehydration [43.4% vs. 27.1%, p = 0.001] at hospital admission and a higher frequency of stage 1 AKI [94.2% vs. 87.0%, p = 0.044]. Individuals with H-AKI needed more often mechanical ventilation [30.7% vs. 19.6%, p = 0.012] and intensive care [39.1% vs. 26.5%, p = 0.009] and had higher in-hospital mortality [31.3% vs. 19.0%, p = 0.006] than those with C-AKI. There was no difference in 12-month mortality between C-AKI and H-AKI patients [17.6% vs. 12.9%, respectively, p = 0.267] (Table 3).

**Table 3:** Comparison between patients with community-acquired and hospital-acquired acute kidney injury.

Variable	C-AKI (n = 189)	H-AKI (n = 192)	p value
Age, years	62.0 (4837–73.6)	62.4 (90.0 (72.8)	0.988
Male sex, % (n)	40.2 (76)	50.0 (96)	0.055
Comorbidities			
Arterial hypertension, % (n)	52.9 (100)	51.0 (98)	0.715
Diabetes mellitus, % (n)	31.7 (60)	29.2 (56)	0.584
Heart failure, % (n)	24.9 (47)	31.3 (60)	0.166
Chronic liver disease, % (n)	22.8 (43)	17.7 (34)	0.220
CKD, % (n)	15.3 (29	14.6 (28)	0.835
Number of comorbidities	2 (1–3)	2 (1–3)	0.937
Causes of hospitalization			
Respiratory system diseases, % (n)	28.0 (53)	33.9 (65)	0.128
Diseases of the genitourinary tract, % (n)	**23.3 (44)**	**13.5 (26)**	
Diseases of the cardiovascular system, % (n)	6.3 (12)	12.0 (23)	
Infection/sepsis, % (n)	12.2 (23)	9.9 (19)	
Liver/bile duct diseases, % (n)	10.1 (19)	9.4 (18)	
Previously used medications			
ACEI or ARB, % (n)	32.1 (60)	27.8 (52)	0.366
Nonsteroidal anti-inflammatory drugs, % (n)	0.0 (0)	2.5 (4)	0.136
Physical examination on admission			
Arterial hypotension, % (n)	**24.3 (46)**	**12.5 (24)**	**0.003**
Dehydration, % (n)	**43.4 (82)**	**27.1 (52)**	**0.001**
Edema, % (n)	15.3 (29)	20.3 (39)	0.205
Stages of AKI			
1, % (n)	**94.2 (178)**	**87.0 (167)**	**0.044**
2, % (n)	4.2 (8)	7.8 (15)	
3, % (n)	1.6 (3)	5.2 (10)	
Use of vasopressors, % (n)	17.5 (33)	25.0 (48)	0.072
Need for mechanical ventilation, % (n)	**19.6 (37)**	**30.7 (59)**	**0.012**
Need for dialysis, % (n)	3.7 (7)	6.3 (12)	0.254
Nonrenal outcomes			
Need for ICU, % (n)	**26.5 (50)**	**39.1 (75)**	**0.009**
Length of stay (days)	11 (7–18)	10 (5–17)	0.254
In-hospital death, % (n)	**19.0 (36)**	**31.3 (60)**	**0.006**
Death at 12 months, % (n)	17.6 (27)	12.9 (17)	0.267

Data are presented as % (n) or median (IQR). AKI, acute kidney injury. C-AKI, community-acquired AKI. H-AKI, hospital-acquired AKI. CKD, chronic kidney disease. ICU, intensive care unit. The numbers in bold indicate column values that differ from each other within the same row at the 0.05 significance level.

### Comparing hospital survivor and non-survivor groups

Compared with the survivors, the non-survivors were older [65.9 (54.0–77.6) vs. 58.7 (45.0–70.3) years, p<0.001], had chronic liver disease more often [25.9% vs. 14.9%, p = 0.002], had more comorbidities [2 (1–3) vs. 1 (0–3), p = 0.008], had more hospitalizations due to pulmonary diseases [40.0% vs. 29.9%, p = 0.014], and had fewer hospitalizations due to genitourinary system diseases [8.9% vs. 16.1%, p = 0.014]. At hospital admission, compared with survivors, non-survivors had a higher frequency of hypotension [24.4% vs. 12.9%, p = 0.001], dehydration [43.7% vs. 26.2%, p<0.001], and higher serum creatinine [1.19 (0.77–1.88) vs. 0.95 (0.74–1.33) mg/dl, p = 0.001]. When compared to survivors, the non-survivors had a lower frequency of stage 1 AKI [83.3% vs. 93.0%], a higher frequency of stage 3 AKI [8.3% vs. 1.8%)], a higher frequency of H-AKI [51.9% vs. 25.3%, p<0.001], a similar frequency of C-AKI [29.6% vs. 27.0%, p = 0.539], higher use of vasopressors [61.5% vs. 4.4%, p<0.001], greater need for mechanical ventilation [66.7% vs. 7.0%, p<0.001], more need for intensive care [73.3% vs. 13.4%, p<0.001], and longer hospital stay [8 (4–14) vs. 12 (6–22) days, p<0.001)]. The in-hospital mortality of patients with AKI stage 1, 2 and 3 was 23.2% (80/345), 34.8% (8/23) and 61.5% (8/13), respectively (Table 4).

**Table 4:** Comparison between survivors and non-survivors.

Variable	In-hospital death			Death within 12 months		
	No (n = 596)	Yes (n = 135)	p value	No (n = 512)	Yes (n = 84)	p value
Age, years	**58.7 (45.0–70.3)**	**65.9 (54.0–77.6)**	**<0.001**	**57.6 (42.4–69.2)**	**65.9 (55.9–76.6)**	**<0.001**
Male sex, % (n)	53.2 (317)	62.2 (84)	0.057	**51.4 (263)**	**64.3 (54)**	**0.028**
Comorbidities						
Arterial hypertension, % (n)	47.0 (280)	51.1 (69)	0.386	45.5 (233)	56.0 (37)	0.075
Diabetes mellitus, % (n)	28.5 (170)	23.0 (31)	0.191	28.7 (147)	27.4 (23)	0.802
Heart failure, % (n)	22.3 (133)	27.4 (37)	0.206	**19.5 (100)**	**39.3 (33)**	**<0.001**
Chronic liver disease, % (n)	**14.9 (89)**	**25.9 (35)**	**0.002**	**11.7 960)**	**35.5 (29)**	**<0.001**
CKD, % (n)	10.4 (62)	10.4 (14)	0.991	9.6 (46)	15.5 (13)	0.100
Number of comorbidities	**1 (0–3)**	**2 (1–3)**	**0.008**	**1 (0–3)**	**2 (1–3)**	<0.001
Causes of hospitalization						**0.035**
Respiratory system diseases, % (n)	**29.9 (178)**	**40.0 (54)**	**0.014**	30.7 (157)	25.0 (21)	
Diseases of the genitourinary tract, % (n)	**16.1 (96)**	**8.9 (12)**		16.0 (82)	16.7 (14)	
CVD, % (n)	10.9 (65)	9.6 (13)		11.3 (58)	8.3 (7)	
Infection/sepsis, % (n)	9.6 (47)	12.6 (17)		9.6 (49)	9.5 (8)	
Liver/bile duct diseases, % (n)	7.9 (47)	11.1 (15)		**6.4 (33)**	**16.7 (14)**	
Parameters at admission						
Arterial hypotension, % (n)	**12.9 (77)**	**24.4 (33)**	**0.001**	13.9 (71)	7.1 (6)	0.089
Dehydration, % (n)	**26.2 (156)**	**43.7 (59)**	**<0.001**	25.0 (128)	33.3 (28)	0.107
Edema, % (n)	14.6 (87)	14.1 (19)	0.876	13.5 (69)	21.4 (18)	0.056
Serum creatinine (mg/dl)	**0.95 (0.74–1.33)**	**1.19 (0.77–1.88)**	**0.001**	**0.93 (0.72–1.29)**	**1.19 (0.85–1.81)**	**<0.001**
eGFR (ml/min/m^2^)	**74.6 (49.1–101.3)**	**54.2 (34.9–90.1)**	**<0.001**	**78.7 (50.6–104.4)**	**59.9 (35.8–79.4)**	**<0.001**
eGFR < 60 ml/min/m^2^, % (n)	**35.1 (209)**	**54.1 (73)**	**<0.001**	**32.6 (167)**	**50.0 (42)**	0.002
Use of vasopressors, % (n)	**4.4 (26)**	**61.5 (83)**	**<0.001**	4.3 (22)	4.8 (4)	0.847
Mechanical ventilation, % (n)	**7.0 (42)**	**66.7 (90)**	**<0.001**	6.6 (34)	9.5 (8)	0.339
Kidney outcomes						
Stages of AKI						
1, % (n)	**93.0 (265)**	**83.3 (80)**	**0.009**	92.5 (223)	95.5 (42)	0.414
2, % (n)	5.3 (15)	8.3 (8)		5.4 (13)	4.5 (2)	
3, % (n)	**1.8 (5)**	**8.3 (8)**		2.1 (5)	0.0 (0)	
Location of the AKI						
AKI-C, % (n)	27.0 (161)	29.6 (40)	0.539	26.0 (133)	33.3 (28)	0.159
H-AKI, % (n)	**25.3 (151)**	**51.9 (70)**	**<0.001**	25.8 (132)	22.6 (19)	0.537
Need for KRT, % (n)	**1.0 (6)**	**13.3 (18)**	**<0.001**	1.0 (5)	1.2 (1)	0.856
Non-kidney outcomes						
Need for ICU, % (n)	**13.4 (80)**	**73.3 (99)**	**<0.001**	13.3 (68)	14.3 (12)	0.802
Length of stay (days)	**8 (4–14)**	**12 (6–22)**	**<0.001**	8 (4–14)	8 (4–13)	0.643

Data are presented as % (n) or median (IQR). CKD, chronic kidney disease. CVD, cardiovascular disease. eGFR, estimated glomerular filtration rate. AKI, acute kidney injury. ICU, intensive care unit. The numbers in bold indicate column values that differ from each other within the same row at the 0.05 significance level.

### Comparing 12 months survivor and non-survivor groups

The non- survivors at 12 months, compared with the survivors, were older [65.9 (55.9–76.6) vs. 57.6 (42.4–69.2) years, p>0.001] and more likely to be male [64.3% vs. 51.4, p = 0.028]; had a higher frequency of heart failure [35.5% vs. 11.7%, p<0.001] and chronic liver disease [39.3% vs. 19.5%, p<0.001]; had more comorbidities [2 [1–3] vs. 1 [0–3], p<0.001]; and had more hospitalizations for liver/bile duct diseases [16.7% vs. 6.4%, p = 0.035]. At hospital admission, compared with survivors, non-survivors had higher serum creatinine [0.93 (0.72–1.29) vs. 1.19 (0.85–1.81) mg/dl, p<0.001)]. There were no significant differences in the need for vasopressors, mechanical ventilation, neither in kidney (AKI stage, AKI site, need for KRT) or non-kidney outcomes (need for vasopressors, ICU admission, length of hospital stay) between survivors and non-survivors at 12 months (Table 4).

### Multivariate analysis results

According to a multiple linear regression, the independent factors associated positively with C-AKI were CKD, chronic liver disease, older age, and negatively was hospitalization for cardiovascular disease. The independent factors associated positively with a greater risk of H-AKI were CKD or heart failure as comorbidities, and hypotension or edema at hospital admission.

The independent factors associated positively with the risk of in-hospital death were age, the presence of dehydration on hospital admission, H-AKI, the use of vasopressors, the need for mechanical ventilation and intensive care. Diseases of the urinary tract were an independent factor associated negatively with in-hospital death. The factors independently associated with a higher risk of death at 12 months were age, heart failure, and chronic liver disease. Female sex was associated with a lower risk of death at 12 months (Table 5).

**Table 5:** Multiple analyses of factors associated with acute kidney injury and death.

Dependent variable	Independent variable	OR (95% CI)	p value
Community-acquired AKI ^1^	Chronic kidney disease	2.32 (1.41–3.82)	0.001
	Chronic liver disease	1.63 (1.07–2.48)	0.023
	Age	1.01 (1.00–1.02)	0.030
	Hospitalization for CVD	0.51 (0.27–0.95)	0.034
Hospital-acquired AKI ^2^	Chronic kidney disease	2.53 (1.35–4.75)	0.004
	Arterial hypotension	2.46 (1.22–4.95)	0.011
	Edema	1.69 (1.01–2.85)	0.048
	Heart failure	1.61 (1.04–2.50)	0.034
In-hospital death ^3^	Use of vasopressor	6.02 (2.85–12.69)	<0.001
	Mechanical ventilation	4.68 (2.17–10.13)	<0.001
	Dehydration	2.92 (1.66–5.16)	<0.001
	ICU admission	2.63 (1.26–5.49)	0.010
	H-AKI	1.88 (1.01–3.52)	0.049
	Age	1.03 (1.01–1.05)	0.002
	Hospitalization due to GUT disease	0.40 (0.17–0.93)	0.034
Death within 12 months ^4^	Chronic liver disease	4.46 (2.54–8.93)	<0.001
	Heart failure	2.86 (1.50–5.46)	0.001
	Age	1.04 (1.02–1.05)	<0.001
	Female sex	0.60 (0.36–0.99)	0.047

OR, odds ratio. CI, confidence interval. H-AKI, hospital-acquired acute kidney injury. ICU, intensive care unit. CVD, cardiovascular disease. GUT, genitourinary tract.

^1^ Model 1 included the following variables: age, diabetes mellitus, chronic liver disease, chronic kidney disease, the use of inhibitors of the renin–angiotensin system or angiotensin receptor blockers, the use of anti-inflammatory drugs, and hospitalizations for cardiovascular or urogenital diseases.

^2^ Model 2 included the following variables: age, sex, arterial hypertension, diabetes mellitus, heart failure, chronic liver disease, chronic kidney disease, mean arterial pressure <65 mmHg, dehydration, and edema.

^3^ Model 3 included the following variables: age, sex, reason for hospitalization was respiratory, hepatobiliary or genitourinary system diseases, mean arterial pressure <65 mmHg, dehydration, estimated glomerular filtration rate at admission <60 ml/min/1.73 m^2^, use of vasopressors, need for mechanical ventilation, intensive care unit stay, hospital-acquired AKI, and community-acquired AKI.

^4^ Model 4 included the following variables: age, sex, heart failure, chronic liver disease, reasons for hospitalization, respiratory and genitourinary system diseases, dehydration, estimated glomerular filtration rate at admission <60 ml/min/1.73 m^2^, use of vasopressors, need for mechanical ventilation, length of stay in the intensive care unit, hospital-acquired AKI, and community-acquired AKI.

## Discussion

The present study shows that AKI was present in more than half of the patients hospitalized at the emergency department of the hospital evaluated. There was a high prevalence of correctable risk factors, such as dehydration and hypotension (44%) among AKI patients. The distribution between C-AKI and H-AKI patients was balanced, and C-AKI patients had better outcomes (ICU requirement and mortality). CKD was the only independent risk factor positively associated with both C-AKI and H-AKI.

The frequency of AKI among patients admitted from the emergency department of the hospital evaluated (52%) was one of the highest observed in studies of the last decade (Table 6S in S1 File) [12, 21–33]. The observed incidence of H-AKI (26%) was similar to that of a Brazilian study conducted only with critically ill patients, a scenario in which the risk of this condition is known to be higher than that among general hospitalizations [7, 12, 22]. There are some possible explanations for this finding. First, the study site was a reference quaternary hospital where the population had a high burden of comorbidities. Many chronic conditions make patients susceptible to AKI [7]. In the study by Ehmann [21], in which the frequency of C-AKI during hospitalization was lower than that in our study (17% vs. 26%), the patients had lower rates of arterial hypertension (31% vs. 48%), diabetes (16% vs. 28%), and heart failure (8% vs. 23%). Similarly, the participants in the study by Scheuermeyer [30], who find a lower incidence of H-AKI than ours (6% vs. 26%), had less arterial hypertension (41% vs. 48%), diabetes (24% vs. 28%), heart failure (9% vs. 23%) and chronic liver disease (9% vs. 17%) than patients in the present study. Second, all patients included in this study underwent at least two serum creatinine measurements, i.e., there was no failure to identify AKI due to the absence of laboratory data, as in retrospective studies [21, 24, 30, 32].

Another explanation for the high frequency of AKI observed in the present study was the recruitment of only patients who were hospitalized, i.e., the denominator for calculating the AKI frequency did not include patients who came to the emergency room but were not admitted to the hospital. In the studies by Foxwell [24] and Jurawan [29], who evaluated all patients seen at the emergency department in the United Kingdom, the prevalence of AKI was 3% and 2%, respectively. We speculate that the time for the patients in this study to be admitted to the high-complexity teaching hospital might contributed to their arrival in worse clinical condition and with a higher risk of AKI. The host institution of this study is public, is a referral center, and has no open access, i.e., patients are transferred from other institutions through the municipal and state regulatory systems. A study conducted in a municipality in the state of São Paulo showed that the waiting time for patient transference may be 27 hours on average in the SUS scenario [34]. It is recognized that the delay in correcting the risk factors for AKI and in applying treatments for the underlying disease can increase the incidence and severity of AKI and raise the risk of death [35–37].

Correctable risk factors for AKI (hypotension and dehydration) at hospital admission in the patients in this study were more prevalent than they were in a study conducted in the Northern Hemisphere [30], but this finding was like the results of a multicenter study conducted in Latin America [25]. This finding could be explained by differences in health and disease determinants among low-, middle-, and high-income countries, such as access to basic sanitation, food, housing, transportation, and education.

Many characteristics of AKI revealed in this Brazilian study reflect the double burden of diseases that affect middle-income and developing countries; that is, infectious-contagious diseases coexist as a public health problem with chronic diseases [38]. In fact, the mean age and comorbidity profile of the patients in this study were similar to those of individuals from developed countries [31]. On the other hand, the high frequency of dehydration and infection during hospitalization is like that in low-income countries [31] (Table 6S in S1 File).

The mortality of participants with AKI in this study (25%) was similar to that of other studies (22–27%) [25, 28, 33]. Patients with C-AKI had a lower ICU need and lower mortality than those with H-AKI, a result that agrees with two systematic reviews [39, 40]. A possible explanation could be that C-AKI in our patients was less severe than H-AKI (we showed a greater proportion of stage 1 AKI in patients with C-AKI than in those with H-AKI) and that C-AKI patients more often had correctable risk factors (such as dehydration) and less severe disease (requirement of mechanical ventilation and ICU admission) than H-AKI patients.

CKD was independently associated with both C-AKI and H-AKI, which is corroborated by current guidelines that recognize this chronic condition as the main susceptibility to AKI, together with advanced age [7]. In fact, a systematic review showed that reduced GFR and proteinuria are independent risk factors for AKI in individuals with or without arterial hypertension or diabetes mellitus [41].

The limitations of this study must be acknowledged. It was not possible to obtain information on kidney function before and after hospitalization to make a detailed assessment of the presence and recovery of AKI. Data on medications used before hospitalization were not available for most patients, which made it impossible to fully assess exposure to nephrotoxic drugs. In addition, we did not have access to the clinical conditions of the patients before admission to the hospital where the study was conducted or to the time at which they might have been unattended before admission.

## Conclusions

AKI was present at admission in more than half of the hospitalizations starting at the emergency department of the hospital evaluated, and there was a high frequency of correctable risk factors such as dehydration and arterial hypotension. C-AKI was less severe and less lethal than H-AKI. CKD was independently associated with C-AKI and H-AKI.

## Supporting information

S1 File Table. (DOCX)

S2 File Dataset. (XLSX)
