# Supplementary material for: Epidemiology of acute kidney injury in the clinical emergency: A prospective cohort study at a high-complexity public university hospital in São Paulo, Brazil
Source: PLoS One. 2024 Sep 5;19(9):e0309949. doi: 10.1371/journal.pone.0309949 (PMC11376543; doi:10.1371/journal.pone.0309949)
Supplement: S1 File — (DOCX) [file pone.0309949.s001.docx]

**Epidemiology of acute kidney injury in the clinical emergency: A prospective cohort study at a high-complexity public hospital in São Paulo, Brazil**

**Supporting information**

**Table 6S. Evaluated studies that addressed the epidemiology of acute kidney injury in the last decade.**

| Author (year) | Location | Drawing | Scenario | Population | Definition of AKI | Patients number | Age | Male sex | Comorbidities | Risk factors for AKI | Frequency of AKI | AKI mortality |
| --- | --- | --- | --- | --- | --- | --- | --- | --- | --- | --- | --- | --- |
| Azevedo (2024, this study) | Brazil | Prospective | Admissions from the DE | Adults | KDIGO^15^ | 731 | 61 | 55% | AH: 48% DM: 28% CKD: 10% CHF: 23% LCD.: 17% | Dehydration: 29% Hypotension: 15% Cardiovascular event: 11%  Infection/sepsis: 10% | AKI-C: 26% AKI-H: 26% | 25% |
| Ehmann^1^ (2023) | USA | Retrospective | Admissions from the DE | Adults | KDIGO^15^ | 178.927 | 58,0% | 43,0% | AH: 31% DM: 16% CKD: 12% CHF: 8% | nd | AKI-C:17% | 16% |
| Inda-Filho^2^ (2021) | Brazil | Prospective | ICU | Adults | KDIGO^15^ | 8.131 | 66 | 48% | AH: 54% DM: 28% CHF: 6% LCD.: 1% | Postoperative: 21% Infection/sepsis:10% | AKI-H: 26% | 26% |
| Patidar^3^ (2020) | USA | Prospective | Admissions from the DE | Adults with cirrhosis | KDIGO^15^ | 519 | 58 | 57% | AH: 25% DM: 21% LCD.: 100% | Hepatic encephalopathy: 26% Digestive bleeding: 19% Ascites: 14% | AKI-C: 25% AKI-H: 10% | 36% |
| Melo^4^ (2020) | Overall | Systematic review | ICU developed countries | Adults | KDIGO^15^ | 487.983 | 62 | nd | DM: 27% | Sepsis: 4-100%  Nephrotoxicity: 7-85%  Shock: 9-46%  Postoperative: 21-59% | AKI: 39% | 31% |
|  |  |  | ICU in developing countries | Adults | KDIGO^15^ | 5.978 | 57 | nd | DM: 25% | Sepsis: 3-100%  Shock:12-75%  Nephrotoxicity: 10-33%  Postoperative period: no data | AKI: 35% | 55% |
| Foxwell^5^ (2020) | United Kingdom | Retrospective | Consultations in the DE | Adults | KDIGO^15^ | 20.241 | nd | 50% | nd | Infection/sepsis: 24%  Cardiovascular event: 10%  Multiple trauma: 8%  Urinary obstruction: 6%  Urgent surgery: 5% | AKI-C: 3% | nd |
| Lombardi^6^ (2019) | Latin America | Prospective | Admissions from the DE | Adults with AKI | KDIGO^15^ | 905 | 64 | 61% | DM: 28% CKD: 30% Anemia: 18%  CHF: 15% LCD.: 6% | Dehydration: 45% Hypotension: 41% Cardiovascular event: 17%  Urinary obstruction: 8%  Contrasts: 5% | nd | 27% |
| Hanson^7^ (2018) | USA | Retrospective | Admissions from the DE | Children and adolescents | KDIGO^15^ | 13.827 | nd | 52% | CHD: 1.3%  Solid organ tumor: 0.8%  Bone marrow transplant: 0.4% | Nephrotoxicity: 17%  Shock/trauma: 13%  Cardiovascular event: 0.4% | AKI-H: 10% | nd |
| Silveira-Santos^8^ (2018) | Brazil | Prospective | Admissions from the DE | Elderly people with AKI | AKIN | 286 | 75 | 55% | AH: 77% DM: 28% CKD: 34% ICC: 25% LCD: 5% | nd | nd | 53% |
| Safari^9^ (2018) | Iran | Retrospective | Admissions from the DE | Adults with AKI | Record in medical records | 770 | 63 | 59% | AH: 46% DM: 16% CHF: 7%  Cancer: 6% | Hypotension: 28%  Urinary obstruction: 11%  Nephrotoxicity: 10%  Rhabdomyolysis: 8% | nd | 22% |
| Jurawan^10^ (2017) | United Kingdom | Retrospective | Consultations in the DE | Adults | KDIGO^15^ | 112.366 | nd | 53% | nd | nd | AKI-C: 2% | - |
| Scheuermeyer^11^ (2017) | Canada | Retrospective | Admissions from the DE | Adults | KDIGO^15^ | 840 | 68 | 55% | AH: 41% DM: 24% KCD: 12% CHF: 9% LCD.: 3% | Sepsis: 16%:  Cardiovascular event: 16%  Dehydration: 11% | AKI-H: 6% | 12% |
| Mehta^12^ (2016) | Overall | Prospective | ED and ICU admissions in high-income countries | Adults and children with AKI | KDIGO^15^ | 1.260 | 63 | 57% | DM: 28% CKD: 26% CHF: 20% LCD.: 7% | Infection/sepsis: 56% Dehydration: 39% Nephrotoxicity: 29% Cardiovascular event: 24% Postoperative period: 9% | AKI-C: 50% | 10% |
|  |  |  | ED and ICU admissions in upper-middle-income countries | Adults and children with AKI | KDIGO^15^ | 1.605 | 64 | 61% | DM: 26% CKD: 23% CHF: 26% LCD.: 9% | Infection/sepsis: 53% Dehydration: 32% Nephrotoxicity: 22% Cardiovascular event: 28% Postoperative period: 7% | AKI-C: 51% | 11% |
|  |  |  | ED and ICU admissions in lower-middle-income and low-income countries | Adults and children with AKI | KDIGO^15^ | 1.153 | 50 | 62% | DM: 31% CKD: 14% CHF: 11% LCD.: 5% | Infection/sepsis: 75% Dehydration: 46% Nephrotoxicity: 23% Cardiovascular event: 13% Postoperative period: 4% | AKI-C: 77% | 12% |
| Challiner^13^ (2014) | United Kingdom | Retrospective | Admissions from the DE | Adults | AKIN^16^ e RIFLE^17^ | 745 | nd | 55% | nd | nd | AKI-C: 9% AKI-H: 16% | 11% |
| Susantitaphong^14^ (2013) | Overall | Systematic review | Admissions from the DE | Adults | KDIGO^15^ | 3.571.691 | nd | 65% | nd | nd | AKI: 22% | 24% |
|  |  |  |  | Children and adolescents | KDIGO^15^ | 14.220 | nd | 56% | nd | nd | AKI: 38% | 14% |

AKI. acute kidney injury. C-AKI, community-acquired AKI. H-AKI, hospital-acquired AKI. AH, arterial hypertension. DM, diabetes mellitus. CKD, chronic kidney disease. CHF, congestive heart failure. LCD, chronic liver disease. DE, emergency department. ICU, intensive care unit. CHD, congenital heart disease. Solid organ transplantation, solid organ transplantation. BMT, bone marrow transplantation. KDIGO, Kidney Disease Improvement Global Outcomes. AKIN, acute kidney injury network. RIFLE, risk, injury, failure, loss, end-stage renal disease. nd, not described.

**References to supplementary material**

1. Ehmann MR, Klein EY, Zhao X, et al. Epidemiology and Clinical Outcomes of Community-Acquired Acute Kidney Injury in the Emergency Department: A Multisite Retrospective Cohort Study. Am J Kidney Dis. 2023; S0272-6386(23)00945-9. doi: 10.1053/j.ajkd.2023.10.009
2. Inda-Filho AJ, Ribeiro HS, Vieira EA, Ferreira AP. Epidemiological profile of acute kidney injury in critically ill patients admitted to intensive care units: A Prospective Brazilian Cohort. J Bras Nefrol. 2021;43(4):580-585. doi: 10.1590/2175-8239-JBN-2020-0191
3. Patidar KR, Shamseddeen H, Xu C, Ghabril MS, et al. Hospital-Acquired Versus Community-Acquired Acute Kidney Injury in Patients With Cirrhosis: A Prospective Study. Am J Gastroenterol. 2020;115(9):1505-1512. doi: 10.14309/ajg.0000000000000670
4. Melo FAF, Macedo E, Fonseca Bezerra AC, et al. A systematic review and meta-analysis of acute kidney injury in the intensive care units of developed and developing countries. PLoS One. 2020;15(1):e0226325. doi: 10.1371/journal.pone.0226325
5. Foxwell DA, Pradhan S, Zouwail S, Rainer TH, Phillips AO. Epidemiology of emergency department acute kidney injury. Nephrology (Carlton). 2020;25(6):457-466. doi: 10.1111/nep.13672
6. Lombardi R, Ferreiro A, Claure-Del Granado R, et al.; EPILAT-ITA Study Group. EPILAT-IRA Study: A contribution to the understanding of the epidemiology of acute kidney injury in Latin America. PLoS One. 2019;14(11):e0224655. doi: 10.1371/journal.pone.0224655
7. Hanson HR, Babcock L, Byczkowski T, Goldstein SL. Describing pediatric acute kidney injury in children admitted from the emergency department. Pediatr Nephrol. 2018;33(7):1243-1249. doi: 10.1007/s00467-018-3909-3
8. Silveira Santos CGD, Romani RF, Benvenutti R, et al. Acute Kidney Injury in Elderly Population: A Prospective Observational Study. Nephron. 2018;138(2):104-112. doi: 10.1159/000481181
9. Safari S, Hashemi B, Forouzanfar MM, Shahhoseini M, Heidari M. Epidemiology and Outcome of Patients with Acute Kidney Injury in Emergency Department; a Cross-Sectional Study. Emerg (Tehran). 2018;6(1):e30
10. Jurawan N, Pankhurst T, Ferro C, Nightingale P, et al. Hospital acquired Acute Kidney Injury is associated with increased mortality but not increased readmission rates in a UK acute hospital. BMC Nephrol. 2017;18(1):317. doi: 10.1186/s12882-017-0729-9
11. Scheuermeyer FX, Grafstein E, Rowe B, et al. The Clinical Epidemiology and 30-Day Outcomes of Emergency Department Patients With Acute Kidney Injury. Can J Kidney Health Dis. 2017; 4:2054358117703985. doi: 10.1177/2054358117703985
12. Mehta RL, Burdmann EA, Cerdá J, et al. Recognition and management of acute kidney injury in the International Society of Nephrology 0by25 Global Snapshot: a multinational cross-sectional study. Lancet. 2016;387(10032):2017-25. doi: 10.1016/S0140-6736(16)30240-9
13. Challiner R, Ritchie JP, Fullwood C, Loughnan P, Hutchison AJ. Incidence and consequence of acute kidney injury in unselected emergency admissions to a large acute UK hospital trust. BMC Nephrol. 2014;15:84. doi: 10.1186/1471-2369-15-84
14. Susantitaphong P, Cruz DN, Cerda J, et al.; Acute Kidney Injury Advisory Group of the American Society of Nephrology. World incidence of AKI: a meta-analysis. Clin J Am Soc Nephrol. 2013; 8(9):1482-93. doi: 10.2215/CJN.00710113
15. Kidney Disease: Improving Global Outcomes (KDIGO) Acute Kidney Injury Work Group. KDIGO Clinical Practice Guideline for Acute Kidney Injury. Kidney inter., Suppl. 2012; 2: 1–138.
16. Mehta RL, Kellum JA, Shah SV, et al.; Acute Kidney Injury Network. Acute Kidney Injury Network: report of an initiative to improve outcomes in acute kidney injury. Crit Care. 2007;11(2):R31. doi: 10.1186/cc5713
17. Kellum JA, Levin N, Bouman C, Lameire N. Developing a consensus classification system for acute renal failure. Curr Opin Crit Care. 2002;8(6):509-14. doi: 10.1097/00075198-200212000-00005
